# Effect of *Pseudomonas moorei* KB4 Cells’ Immobilisation on Their Degradation Potential and Tolerance towards Paracetamol

**DOI:** 10.3390/molecules26040820

**Published:** 2021-02-04

**Authors:** Robert Surma, Danuta Wojcieszyńska, Jagna Karcz, Urszula Guzik

**Affiliations:** Institute of Biology, Biotechnology and Environmental Protection, Faculty of Natural Science, University of Silesia in Katowice, Jagiellonska 28, 40-032 Katowice, Poland; rsurma@us.edu.pl (R.S.); danuta.wojcieszynska@us.edu.pl (D.W.); jagna.karcz@us.edu.pl (J.K.)

**Keywords:** biodegradation, non-steroidal anti-inflammatory drugs, immobilisation, *Pseudomonas*, loofah sponge, paracetamol

## Abstract

*Pseudomonas moorei* KB4 is capable of degrading paracetamol, but high concentrations of this drug may cause an accumulation of toxic metabolites. It is known that immobilisation can have a protective effect on bacterial cells; therefore, the toxicity and degradation rate of paracetamol by the immobilised strain KB4 were assessed. Strain KB4 was immobilised on a plant sponge. A toxicity assessment was performed by measuring the concentration of ATP using the colony-forming unit (CFU) method. The kinetic parameters of paracetamol degradation were estimated using the Hill equation. Toxicity analysis showed a protective effect of the carrier at low concentrations of paracetamol. Moreover, a pronounced phenomenon of hormesis was observed in the immobilised systems. The obtained kinetic parameters and the course of the kinetic curves clearly indicate a decrease in the degradation activity of cells after their immobilisation. There was a delay in degradation in the systems with free cells without glucose and immobilised cells with glucose. However, it was demonstrated that the immobilised systems can degrade at least ten succeeding cycles of 20 mg/L paracetamol degradation. The obtained results indicate that the immobilised strain may become a useful tool in the process of paracetamol degradation.

## 1. Introduction

Among the pharmaceuticals consumed, non-steroidal anti-inflammatory drugs (NSAIDs), such as ibuprofen, naproxen or diclofenac, belong to the most frequently used drugs. The consumption of NSAIDs in developed countries has reached a rate of tonnes per year [1,2]. However, the exact global consumption rates are difficult to calculate because NSAIDs are mostly sold as over-the-counter drugs, including pharmaceutical preparations with different trade names.

NSAIDs and their metabolites belong to a group of the most frequently detected contaminants. Since NSAIDs are bioactive compounds, they pose a threat to living organisms and environmental processes. Among the reasons for their presence in the environment, low removal rates in wastewater treatment plants and improper disposal of unused drugs should be mentioned [3]. Another way for drugs to enter the environment is soil irrigation with recycled water and the application of biosolids as fertilisers [4,5].

More and more effort is being put into developing water and soil remediation techniques for xenobiotic removal. Remediation methods such as the utilisation of granular or powdered activated carbon, nanofiltration, photocatalysis with titanium dioxide, reverse osmosis or membrane bioreactors are constantly being developed, and the number of their applications is growing. Pollutants such as NSAIDs (in parent form or as their metabolites, often more active and/or stable) are, however, incessantly being released into the environment for reasons such as the lack of optimal treatment techniques [6]. Bioremediation of contaminated groundwater or soil is considered to be a relatively environmentally friendly, simple and cheap tool for removing xenobiotics from the environment. The method mostly utilises naturally occurring xenobiotic-degrading microbes. The number of reported microorganisms capable of NSAID degradation is constantly increasing. NSAID-degrading bacteria are a non-homogeneous group, and reported examples belong to a broad spectrum of different genera such as *Klebsiella*, *Delftia*, *Patulibacter*, *Stenotrophomonas*, *Pseudomonas*, *Labrys*, *Raoultella*, *Brevibacterium*, *Planococcus*, *Bacillus*, *Enterobacter*, *Sphingomonas*, *Pseudaminobacter*, *Ralstonia* and *Streptomyces* [7,8,9,10,11,12,13,14,15,16,17,18,19,20].

*P. moorei* KB4 is one of the few described bacterial strains capable of degrading either paracetamol (4-hydroxyacetanilide, N-acetyl-p-aminophenol, acetaminophen), an analgesic and antipyretic drug often described as an NSAID, or diclofenac, an NSAID generally accepted as being dangerous for the environment [21]. The main products of paracetamol degradation by KB4 were identified as p-aminophenol and hydroquinone. *P. moorei* KB4 is able to degrade paracetamol in the presence of harmful co-pollutants such as heavy metals, phenol and its chlorinated derivatives [22]. One way to improve the efficiency of the degradation process is to immobilise the microorganisms involved. Immobilisation has been reported to promote bioremediation processes and to allow for the use of multiple biocatalysts. It is also known to decrease the toxic effect of xenobiotics against the immobilised bacteria and improve their resistance to harmful environmental factors. Cell adsorption on the surface of the carrier is the most popular method of immobilisation due to its simplicity and non-toxicity [23]. At the early stages of the immobilisation process, the cells’ attachment is reversible, and they may be easily washed from the carrier. Afterwards, their binding to the surface of the carrier becomes stronger due to the synthesis of extracellular polymeric substances. It is desirable that the formed biofilm should be strongly bound to the carrier. This feature depends on the properties of a given microorganism and the type of the surface, but the quality of the formed biofilm may also be improved by selecting optimal conditions of immobilisation [24,25]. *P. moorei* KB4 exhibits qualities that facilitate whole-cell immobilisation: a high self-aggregation index and a strong biofilm formation ability under certain conditions. It has been demonstrated to be capable of being adsorbed by carriers and utilised in paracetamol degradation [21]. The loofah sponge (*Luffa aegyptiaca*) has been chosen as the carrier in the present study. It is used in microorganism immobilisation for its high porosity (85–95%) with simultaneously low density (0.018–0.05 g/cm^3^). The sponges are composed of fibre networks that form good conditions for cell adsorption and biofilm development [26,27].

Strain KB4, with its ability to utilise a wide range of carbon and nitrogen sources and its high tolerance towards various xenobiotics, has been selected for the immobilisation and degradation studies presented in this paper. It is also documented that strain KB4 retains its activity at lower temperatures and may, therefore, be applied in freshwater, marine and other environments [22]. The process of immobilisation has been optimised, and toxicological tests based on colony-forming units (CFU) calculation and ATP determination were performed to compare the toxicity of paracetamol against free and immobilised cells. Degradation tests were performed to calculate the kinetic parameters of biodegradation, if applicable. An additional carbon source may accelerate the degradation of xenobiotics; therefore, the ability of either free or immobilised *P. moorei* KB4 cells to degrade paracetamol in co-metabolic systems with glucose as a carbon source was also evaluated. Additionally, the cyclic supplementation of paracetamol doses was applied to examine the stability and durability of the system consisting of the loofah sponge as a carrier and attached cells.

## 2. Results and Discussion

### 2.1. Optimisation of the Immobilisation Process

**Figure 1:**
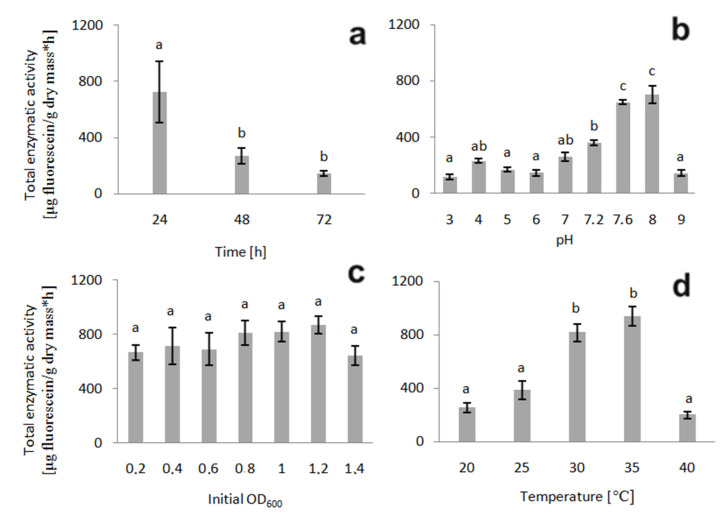
Effect of four different parameters ((**a**)–incubation time; (**b**)–pH; (**c**)–initial OD_600_; (**d**)–temperature) on the metabolic activity of the immobilised *P. moorei* KB4. Total enzymatic activity was considered as a marker of the most favourable conditions. Error bars were obtained based on the standard deviation. Statistically significant differences are marked with letters (post-hoc Tukey HSD (Honestly Significant Difference), *p* ≤ 0.05).

The procedure of *P. moorei* KB4 immobilisation on the loofah sponge through its adsorption on the surface was developed by optimising each parameter separately to obtain the optimal value of the separate parameters in the following order: time, pH, initial OD_600_ and temperature. Commercially available sponges (*Luffa aegyptiaca*-derived) are composed of cellulose, hemicellulose and lignin. This carrier is eco-friendly for bacterial cell immobilisations due to their high mechanical resistance and high porosity [28]. After the optimisation of the selected parameter, its value was used when examining the next one. This intuitional optimisation approach is simple and relatively efficient. A similar method was applied for optimising the immobilisation of *Bacillus thuringiensis* B1(2015b) and *Planococcus* sp. S5, both on loofah sponges as the carrier [25,28]. The optimal values for *P. moorei* KB4 immobilisation presented in Figure 1 (incubation time 24 h, pH 8, initial OD_600_ 1.2 and temperature 35 °C) partially correspond to those found for the two mentioned strains. Identical optimal pH values for *Bacillus thuringiensis* B1(2015b) and initial OD_600_ values for *Planococcus* sp. S5 were obtained in earlier studies [25,28].

Additionally, the final value of the total enzymatic activity of strain KB4 is of the same range of magnitude as for *Bacillus thuringiensis* B1(2015b) [28]; however, it is slightly higher. In contrast to the two other mentioned strains, KB4 forms the most active biofilms at higher temperatures. There seems to be differentiation among bacteria, even belonging to the same genera. For instance, certain *Salmonella enterica* strains tend to produce a biofilm at temperatures <20 °C (with optimal pH depending on the surface material) [29]. In contrast, a different study demonstrated that most *Salmonella* strains prefer high temperatures (37 °C) to form biofilms [30]. *P. moorei* KB4 has been previously immobilised on bacterial cellulose disks produced by the *Komagataeibacter xylinus* E-89370 strain, and the mineral salts medium (MSM) was chosen for the immobilisation assay. In the same study, the effect of different media on the biofilm formation was tested, and the best results were obtained for MSM with the pre-cultivation in Sutherland–Wilkinson medium [21]. The MSM was chosen for all experiments (the step of pre-cultivation in Sutherland–Wilkinson medium was omitted to simplify the procedure and reduce the potential application costs), either concerning immobilisation, degradation or toxicity. The limitation of a carbon source in some cases inhibits the biofilm formation, while sometimes resulting in a stimulatory effect. For instance, *Staphylococcus aureus* and *Staphylococcus epidermidis* form biofilms only when glucose, a substrate for adhesin synthesis, is present in the medium [31]. On the other hand, the lack of an additional carbon source was reported to stimulate biofilm formation by *Bacillus subtilis*, which is produced to provide conditions sufficient for survival and proliferation during nutrient deficiency [32]. Another example of a strain preferring a rich immobilisation medium is the *Bacillus thuringiensis* B1(2015b) mentioned above, which was found to immobilise on loofah sponges most efficiently in the sporulation-specific medium (HCT). The use of a rich immobilisation medium is probably the reason for the enzymatic activity along with the B1(2015b) immobilisation time [28]. In the present study, where KB4 cells were lacking any carbon sources during immobilisation, the total enzymatic activity decreased with time, even though the biofilm mass increased, which indicates efficient adsorption of cells to the support. However, non-growth conditions (no easily degradable carbon source) resulted in decreased total enzymatic activity due to cell viability loss. The total enzymatic activity measurement resulted in recording 24 h as the optimal immobilisation time to produce a highly active biofilm (Figure 1). This result is not in contradiction to Żur et al. [21], classifying strain KB4 as a weak biofilm producer after 24 h of incubation, since the classification was made following the method developed by Stepanović et al. [33] that measures the biofilm quantity, not its activity.

**Figure 2:**
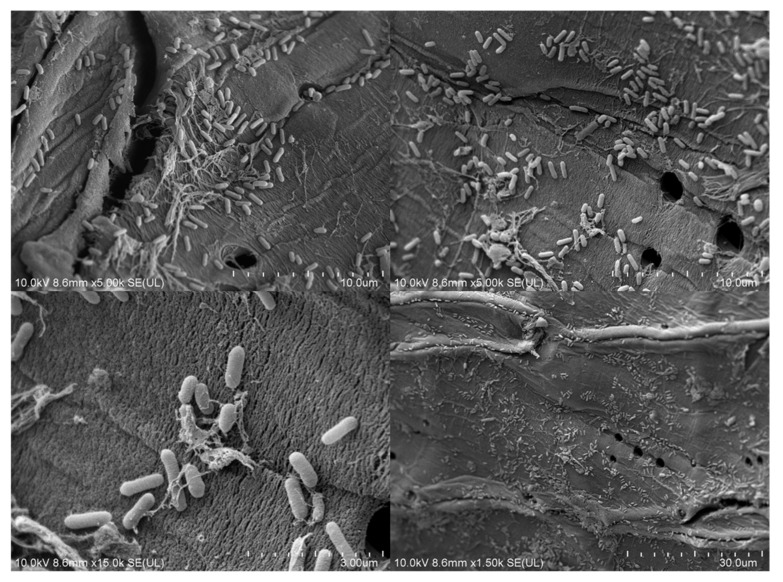
SEM micrographs of *P. moorei* KB4 cells immobilised in optimal conditions. The working distance varies between 3 and 300 μm.

Fragments of the carrier with attached bacterial cells (immobilisation was conducted in optimal conditions) were observed in the SEM. SEM analysis confirmed the successful cell attachment to the carrier (Figure 2). The structures observed may be described as flat, monolayer biofilms forming irregular bands of different sizes. The porous structure of the carrier was also displayed. The observations are similar to those resulting from the SEM analysis of immobilised *Bacillus thuringiensis* B1(2015b) and *Planococcus* sp. S5 [25,28].

### 2.2. Degradation Studies

Previous research has shown that *P. moorei* KB4 is strain that can degrade up to 50 mg/L of paracetamol within a few hours and transform diclofenac [22]. In contrast, earlier studies with a free strain did not show its ability to degrade ibuprofen and naproxen (data not published). Because immobilisation may affect the metabolism of bacterial cells [34], in the present studies, it was checked whether the immobilised strain KB4 would break down ibuprofen. Unfortunately, the immobilised strain KB4 was only capable of degrading paracetamol, while ibuprofen remained unaffected. For this reason, in further steps, only the effect of immobilisation on the kinetic degradation and toxicity of paracetamol was investigated.

Ribeiro et al. [35] tested the ability to adsorb paracetamol on the *Luffa cylindrica* plant sponge. However, in their research, the sponge was fragmented (particles 4.7 mm in size) and packed in the column together with sand and gravel, which increased the adsorption capacity. Despite such conditions, the authors obtained a maximum of 40% paracetamol adsorption at its low concentration (5 µM). Moreover, in their research, they did not determine the actual percentages of paracetamol that were adsorbed on the sponge and the sand and gravel. In our study, we did not find adsorption of paracetamol on the sponge. There was no loss of paracetamol in the abiotic control, which clearly indicates its biological degradation.

**Figure 3:**
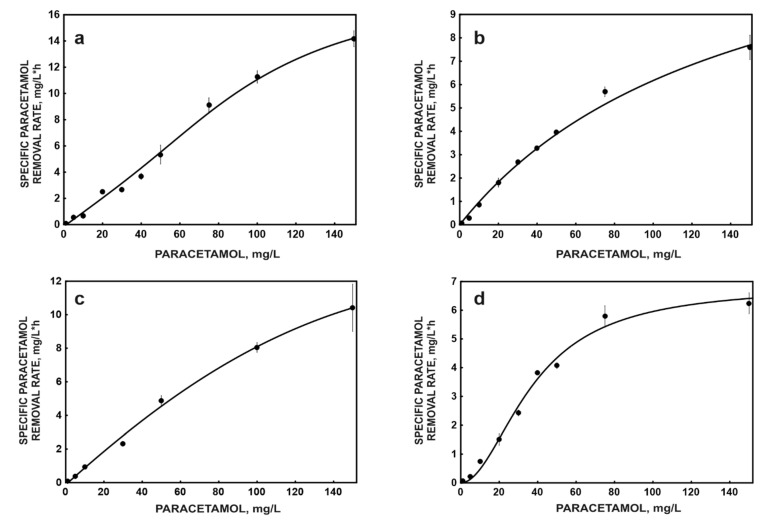
Kinetic models of paracetamol degradation by free (**a**,**c**) and immobilised (**b**,**d**) cells of *P. moorei* KB4 in the mono-substrate system (**a**,**b**) and the co-metabolic system with glucose as a growth substrate (**c**,**d**). The data points represent the average of three independent experiments.

The Monod model is often used to compare the degradation kinetics of free and immobilised cells. This model describes the relationship between the degradation rate of a substrate and its initial concentration when the linear increase in the degradation rate starts at a value of zero [36,37,38]. However, the collected data indicated that in the systems with free cells without glucose and immobilised cells with glucose, there was a delay in degradation (Figure 3). Many sigmoidal models can be found in the literature, such as the Gompertz, Richards and Stannard models and the logistics model. However, most of the equations that describe sigmoid curves only contain mathematical parameters, not parameters of biological importance, making it difficult to determine the kinetic constant of biological processes [39]. We decided to use the Hill model used in enzymology to estimate the kinetic parameters in these systems (Table 1).

The obtained kinetic parameters and the course of the kinetic curves (Figure 3) clearly indicate a decrease in the degradation activity of cells after their immobilisation. The reason for this may be because access to the substrate is difficult. The higher degradative activity of free cells in the presence of glucose is a frequently observed effect in co-metabolic systems. The presence of an easily digestible growth substrate contributes to faster growth of biomass, the synthesis of cofactors necessary for substrate decomposition and the induction of degradation enzymes. A similar effect was observed in the presence of sucrose during caffeine degradation by *Fusarium solani* [40]. On the other hand, there are known examples where glucose improves the xenobiotic degradation rate only in low concentrations, when higher co-substrate concentrations decrease the xenobiotic degradation rate. This has been demonstrated, for instance, for different phenol-degrading bacteria [41]. This may happen if the easily degradable co-substrate occurs in abundance [42]. It is possible that the glucose decreased the V_max_ of paracetamol degradation of the immobilised strain KB4 in that manner, while free cells were in a different metabolic state and V_max_ was increased by glucose. The presence of a growth substrate significantly increases the K_s_ constant, which clearly indicates a decrease in the affinity of cells for the substrate. However, the presence of a growth substrate significantly increases the K_s_ constant, which clearly shows a reduction in the affinity of cells for the substrate (Table 1). The change in the shape of the curve in the presence of glucose from sigmoid to hyperbolic (Figure 3) may indicate faster synthesis and binding of regulatory molecules at the allosteric sites of degradation enzymes. As a result, it leads to full enzyme activity being obtained in a shorter time and, consequently, to the abolition of the cooperation effect. The opposite effect was observed for immobilised cells. This indicates the molecular and biochemical changes in the biodegradation process forced by binding the cells to the carrier.

Previous studies [21] demonstrated that *P. moorei* KB4 immobilised on the bacterial cellulose network was able, in the presence of glucose, to degrade 150 mg/L of paracetamol in three cycles of 50 mg/L each with very similar degradation rates of 14.35 ± 0.096 mg/L*h. This study demonstrated that the immobilised systems can degrade at least ten succeeding cycles of 20 mg/L. Little is known about the kinetic parameters of paracetamol degradation by any other bacterial strains. In some cases, cell immobilisation clearly improved the efficiency of the xenobiotic degradation process [36,43]. Still, many reports show that immobilised cells degrade a given substance slower, but other aspects of the process, such as inhibitor tolerance, are improved [44].

### 2.3. Toxicity of Paracetamol Towards P. moorei KB4

The toxicity of paracetamol against planktonic and immobilised cells was assessed with the use of two different methods, either directly (CFU) or indirectly (ATP concentration measurement). The results are demonstrated in Table 2 and Table 3.

**Figure 4:**
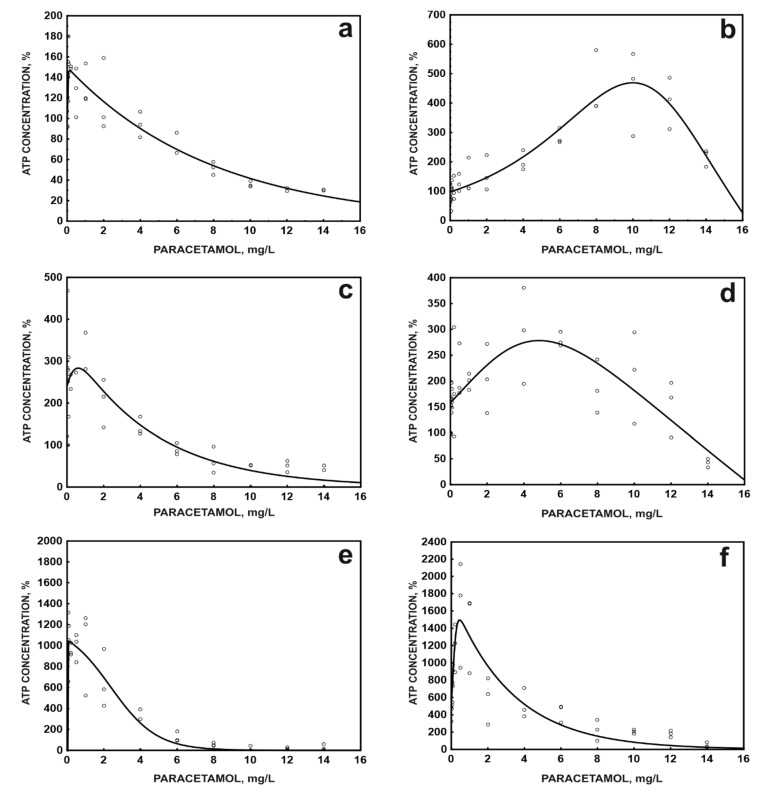
Changes in ATP concentration in free (**a**,**c**,**e**) and immobilised (**b**,**d**,**f**) cells of *P. moorei* KB4 in the presence of paracetamol at various concentrations, after 2- (**a**,**b**), 4- (**c**,**d**), and 24-h exposure (**e**,**f**). The data points represent the average of three independent experiments.

The toxicity analysis by the ATP method showed a protective effect of the carrier at low concentrations of paracetamol. Simultaneously, a pronounced phenomenon of hormesis was observed in the immobilised systems (Figure 4) [45]. Hormesis is defined as a process in a cell or organism that exhibits a biphasic dose response to an environmental factor, characterised by stimulation in low concentration ranges and inhibition at high doses. The hormetic dose response of bacteria has been observed many times, especially in the response of bacterial cells to the presence of metals [46,47,48].

The hormesis effect observed in this study probably results from the earlier contact of *P. moorei* KB4 cells with this drug, which induced not only degradation enzymes but also the enzymes of antioxidant systems, which prevent oxidative damage to cells observed in the presence of NSAIDs [12]. Toxicity testing after exposing bacterial cells to paracetamol for 24 h indicated an increase in the sensitivity of free cells to paracetamol, while immobilised cells did not show a significant change in sensitivity to paracetamol after this exposure time (Figure 4). This confirms previous studies’ conclusions that the hormetic response is time-dependent and transient [47]. A significant increase in cell sensitivity may be related to the response of cells to toxic metabolites appearing during the degradation of paracetamol. It seems that the immobilisation of cells protects them against the formation of aminophenol or hydroquinone, both reported to be toxic towards living organisms, including bacteria [21,49,50,51].

**Figure 5:**
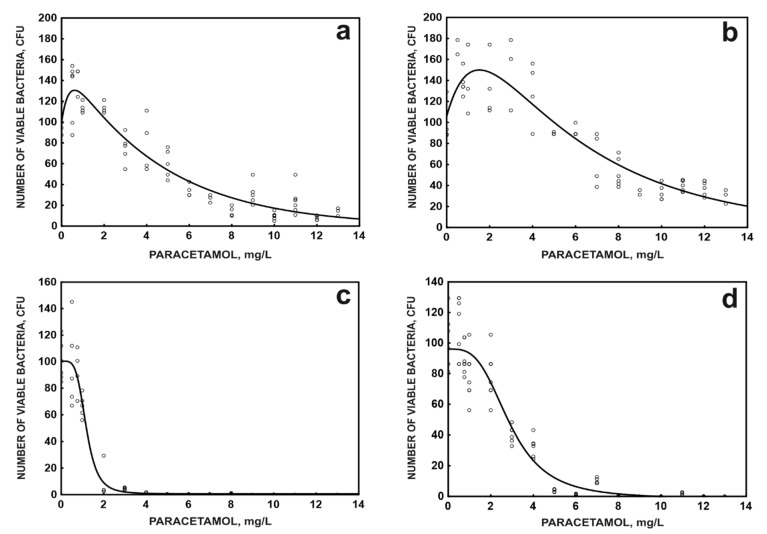
Changes in the number of viable bacteria in free (**a**,**c**) and immobilised (**b**,**d**) cells of *P. moorei* KB4 in the presence of paracetamol (**a**,**b**) or paracetamol and glucose (**c**,**d**). The data points represent the average of three independent experiments.

The analysis of the effect of paracetamol on free and immobilised cells after 24 h of exposure with the CFU method confirmed the results obtained with the ATP method. Additionally, in paracetamol toxicity studies using the CFU method, the paracetamol and glucose system was introduced for comparison. The presence of glucose clearly increases the toxicity of paracetamol to both free and immobilised cells (Figure 5a–d), and the phenomenon of hormesis is also not observed (Figure 5c–d).

This enhancement of toxicity is very surprising. Moreover, the effect is significantly higher in the case of immobilised cells. Glucose is rather known for being a protective factor for bacteria [52] but has been rarely demonstrated to intensify the negative effect of other substances—for instance, it increases the level of oxidative stress caused by other NSAIDs and diclofenac [53].

The presence of detectable concentrations of paracetamol and, more importantly, its toxic metabolites in sewage treatment effluences makes it, potentially, an environmental risk. The Environment Agency (EA) of England and Wales proposed a system ranking the top 10 compounds in terms of the environmental risk they possess. Paracetamol has been classified in fifth place [54,55]. Therefore, more effort needs to be put in to research the toxicity of paracetamol and its derivatives.

## 3. Materials and Methods

### 3.1. Optimisation of P. moorei KB4 Immobilisation

#### 3.1.1. Cell Immobilisation

Cell immobilisation and cell activity measurements were performed in accordance with Dzionek et al. [56], with modifications. Loofah sponges (York, Bolechowo, Poland) were dried in a desiccator to establish a constant weight. The sponges were subsequently cut into fragments weighing 0.15 ± 0.01 g. The obtained cubes were sterilised (121 °C, 1.2 atm). Cells of strain KB4 were immobilised through their adsorption on the surface of a loofah sponge using the natural ability of this strain to form a persistent biofilm. Before immobilisation, the strain was cultivated in a lysogeny broth medium (LB, BTL, Poland; medium composition: peptone k 10 g/L, NaCl 10 g/L, yeast extract 5 g/L) for 24 h under shaking conditions (130 rpm) at 30 °C. Next, the bacterial cultures were centrifuged (5000× *g*, 20 min, 4 °C) and resuspended in a fresh mineral salts medium (MSM) composed of 3.78 g/L Na_2_HPO_4_ × 12H_2_O, 0.5 g/L KH_2_PO_4_, 5.0 g/L NH_4_Cl, 0.2 g/L MgSO_4_ × 7H_2_O and 0.01 g/L yeast extract. Immobilisation was conducted in 250-mL Erlenmeyer flasks, which contained ~0.75 g (5 cubes) of the carrier and 100 mL of the MSM (different pH settings of the medium were tested to find the optimal conditions; tested pH values were 3, 4, 5, 6, 7, 7.2, 7.6, 8 and 9) with *P. moorei* KB4 cells (different initial optical density settings were tested, and measurement was conducted at 600 nm with a Genesys 20 spectrophotometer, Thermo Scientific, Waltham, Massachusetts USA. The following initial optical density settings were tested: 0.2, 0.4, 0.6, 0.8, 1, 1.2 and 1.4). Flasks were incubated under shaking conditions (130 rpm) at different temperature settings (20, 25, 30, 35 and 40 °C) and for different time intervals (24, 48 and 72 h) to find the optimal conditions. Parameters were optimised in the following order: time, pH, initial OD600 and temperature. After optimising each parameter, its optimal value was kept for the rest of the experiment. After the incubation, the loofah sponges with immobilised bacteria were rinsed three times with phosphate-buffered saline (PBS) (pH 7.2).

#### 3.1.2. Total Enzymatic Activity Measurement

Total enzymatic activity was measured by methods with fluorescein diacetate (3′,6′-diacetyl-fluorescein; FDA). This compound is a prefluorophore and is hydrolysed by a broad spectrum of non-specific extracellular enzymes and membrane-bound enzymes such as proteases, lipases and esterases. The hydrolysis product fluorescein has a yellow-green colour and is characterised by strong light absorption at 490 nm. For this reason, the concentration of fluorescein after enzymatic reactions can be measured spectrophotometrically [56]. The dry mass of the immobilised bacterial cells was obtained by comparing the dried weight of the immobilised carrier (105 °C, 2 h and stored in a desiccator) with unimmobilised carrier cubes incubated and dried under the same conditions. The total enzymatic activity of the immobilised cells was measured by adding the rinsed carrier with immobilised bacteria (1 cube) to 8 mL of phosphate-buffered saline (pH 7.2) and incubated for 15 min under shaking conditions (130 rpm, 30 °C). After the pre-incubation, 0.1 mL of FDA (Sigma-Aldrich, St. Louis, MO, USA) (4.8 mmol/L) was slowly injected directly into the middle of the carrier, and the mix was incubated in the dark under shaking conditions (130 rpm, 30 °C) for 1 h. The colouration intensity of the liquid was measured spectrophotometrically at 490 nm. The concentration of fluorescein was calculated based on a standard curve. Total enzymatic activity was expressed as μg fluorescein/g dry mass * h. It was assumed that the highest rate of total enzymatic activity reflects the most optimal immobilisation conditions.

### 3.2. NSAID Degradation Experiments

#### 3.2.1. Degradation Tests

After immobilisation in the optimal conditions established in the previous step, the immobilised cells were washed three times with phosphate-buffered saline. Decomposition tests of selected NSAIDs were conducted in 250-mL Erlenmeyer flasks containing 100 mL of the MSM (pH 7.2) and 5 cubes of the loofah sponge (0.75 g) colonised by bacteria. Each flask was supplemented with either paracetamol or ibuprofen (Sigma-Aldrich, St. Louis, MO, USA). A pilot ibuprofen degradation experiment was performed with an initial concentration of 15 mg/L. Additionally, the same degradation tests were performed with 1 g/L glucose as a co-substrate. Paracetamol degradation experiments were performed either as mono-substrate degradation tests or with 1 g/L glucose as a co-substrate. Initial concentrations of the paracetamol line-up were as follows: 1, 5, 10, 20, 30, 40, 50, 75, 100 and 150 mg/L. Flasks were incubated under shaking conditions (130 rpm at 30 °C). Medium samples were taken in different time intervals (depending on the NSAID being tested, see the Results section), centrifuged (10,000 rpm, 15 min) and analysed using high-performance liquid chromatography (HPLC). The same series of degradation experiments were conducted to compare the kinetic parameters of the immobilised and free cells, but immobilised bacteria were replaced with planktonic cells. The post-immobilisation MSM containing free cells was centrifuged (5000× *g*, 20 min, 4 °C), the supernatant was discarded and cells were resuspended in a small amount of MSM (pH 7.2). Afterwards, the cell suspension was mixed with fresh MSM (pH 7.2) in 250-mL Erlenmeyer flasks to reach a final volume of 100 mL and initial OD_600_ = 0.11. The initial OD_600_ value was established based on the standard curve, demonstrating the dependency between OD_600_ and the dry mass of cells. It was assumed that the initial mass of free cells should be equal to the mass of cells immobilised on 0.75 g of the carrier (0.033 g) in optimal conditions. The standard curve was prepared using 0.2-μm Nuclepore filters, and 10 mL of cell suspension of given OD_600_ values was filtered on a previously dried (105 °C, 2 h) and weighed filter. Afterwards, filters were dried and weighed again. Additionally, immobilised cells were tested in multiple paracetamol application tests. The experiment was conducted as described above (only with 1 g/L glucose as a co-substrate), but each flask was supplemented with a paracetamol dose every 24 h to reach the concentration of 20 mg/L. Medium samples for further analyses were taken at a time interval of 2 h after applying each paracetamol dose.

Additional abiotic controls (medium with paracetamol and sponge without bacteria) were also prepared to determine adsorption or/and abiotic degradation of the drug.

#### 3.2.2. Analysis of NSAID Concentrations

Decomposition rates of NSAIDs used in degradation tests were determined with the HPLC technique using Merck-Hitachi HPLC reversed-phase chromatograph equipped with Ascentis Express ^®^ C18 HPLC Columns (150 × 4.6 mm^2^ for the evaluation of paracetamol concentrations and 100 × 4.6 mm^2^ for ibuprofen), pre-columns Opti-Solw^®^ EXP and a UV/VIS diode array detector. The mobile phase for the ibuprofen assay consisted of acetonitrile and 1% acetic acid (5:95 *v*/*v*) with a flow rate of 1 mL/min. For the paracetamol assay, the mobile phase consisted of methanol and 1% acetic acid (2:98 *v*/*v*) with a 0.7-mL/min flow rate. The detection wavelength for both assays was set at 240 nm [15,22]. NSAID concentrations were identified by comparing the HPLC retention times and UV–visible spectra with those of the external standards. Initial reaction speed values were calculated and used to fit the parameters of the Hill equation for non-hyperbolic regression. The Hill equation is expressed as:

V = V_max_ S^h^/(K_S_ + S^h^), (1)

where v is the specific degradation rate, V_max_ is the maximum specific degradation rate of the substrate, K_S_ is the half-saturation rate constant (mg/L), S is the concentration of the substrate (mg/L) and h is the cooperation coefficient (h > 1-positive cooperation, h < 1-negative cooperation, h = 1 non-cooperation).

### 3.3. Acute Toxicity Assessments

#### 3.3.1. Colony-Forming Units (CFU) Calculation

After determining the optimal conditions for cell immobilisation, either free or immobilised cells were placed in 250-mL Erlenmeyer flasks containing 100 mL of the MSM (pH 7.2) as described in Section 3.1.1. The media were supplemented with different concentrations of paracetamol. Since strain KB4 is able to grow in the mono-substrate culture with paracetamol being the only carbon source [21], the toxicity of paracetamol was tested either in the presence of 1 g/L glucose as a co-substrate or without glucose. After 24 h, serial dilutions of free cell suspension were spread on LB agar plates to estimate the CFU number after overnight incubation. Carrier fragments were previously washed three times in fresh MSM. The pieces of the carrier were then added to 10 mL of fresh MSM and treated in the ultrasonic cleaner for 0.5 min before the serial dilutions step. The inhibitory effects were demonstrated in a manner described in Section 3.2.1.

#### 3.3.2. ATP Determination

Immobilised and free cells were examined in terms of ATP concentrations after exposure to different concentrations of paracetamol. ATP determination is an indirect method that may be used to estimate the viability of biofilm-forming cells [57]. The method was used to complement CFU data.

Carrier fragments with immobilised *P. moorei* KB4 cells were washed three times with fresh MSM. The ATP measurement was performed as follows: the fragment of the carrier was added to 10 mL of fresh MSM containing a specified concentration of paracetamol. The ATP concentration was measured for samples collected after 2, 4 and 24 h of exposure. Samples were treated in the ultrasonic cleaner for 1 min and homogenised for 20 s at 11,000 rpm. The cell viability was estimated using the bioluminescent method based on the oxidation of D-luciferin by firefly luciferase, which occurs in the presence of ATP and is accompanied by fluorescence, the intensity of which is proportional to the ATP content. The ATP concentration was measured immediately after the homogenisation using ATP Determination Kit reagents (Thermo Fisher Scientific, Waltham, MA, USA) according to the protocol provided by the producer. The luminescence was measured with a Spark^®^Multimode Microplate Reader (Tecan, Männedorf, Switzerland). For determination of the viability of the free cells, instead of a carrier cube, an equivalent of post-immobilisation medium with free cells containing an equal ATP concentration compared to a non-exposed control carrier fragment was used. Concentrations were calculated with the use of a calibration curve. Cell response curves were plotted, and adequate ICx values were calculated using the online tool MyCurveFit (https://mycurvefit.com/; access: 2020-11-12) for regular, sigmoidal curves. In the case of irregular response curves, where the hormesis effect was clearly visible, the following function was used as a drug–response model [58]:

y = (a − b/( 1 + exp( c × x − d)))/(1 + exp( e × x − f)),(2)

where x is the logarithm of paracetamol concentration (g/L), and y is the bacterial cell response.

The parameters were estimated using the quasi-Newton algorithm with a varying number of iteration steps and different initial parameter values. Different set-ups were tested manually to fit the equations’ parameters, and each time, the goodness of fit was evaluated as the result of the R values and the linearity of the predicted and observed values. In the case of irregular response curves, ICx values were calculated by solving the adequate equations, where y was replaced with the desired response value.

### 3.4. Carrier Analysis by Scanning Electron Microscopy

To confirm the presence of the attached cells on the carrier surface, scanning electron microscopy (SEM) was used. Fragments of the carrier were prepared for imaging with SEM using 3% *v*/*v* glutaraldehyde incubation (fixative, 24 h) and subsequent ethanol dehydration (30, 50, 70, 80, 90, 95 and 100% *v*/*v*, each for 15 min). The samples were subsequently critical-point dried in the Pelco CPD2 apparatus (Ted Pella Inc., Redding, CA, USA), mounted on aluminium stubs with double-sided adhesive carbon-tape and sputter-coated in a Pelco SC-6 Sputter Coater (Ted Pella Inc., Redding, CA, USA) with a thin film of gold to improve the electrical conductivity of the sample surface. After processing, samples were imaged using the Hitachi SU8010 field emission scanning electron microscope (FESEM) (Hitachi High-Technologies Corporation, Tegama, Japan) at 5, 10 and 15 kV accelerating voltage with a secondary electron detector (SED) and at a working distance (WD) of 3–300 μm.

### 3.5. Statistical Analyses

All experiments were performed in at least three replicates. The values of the total enzymatic activity were analysed using the STATISTICA 12 PL software package. Statistically significant differences and similarities were demonstrated by the post-hoc Tukey HSD test (*p* ≤ 0.05). The Hill equation parameters that were used as the degradation model were estimated using online tool Biomodel (http://biomodel.uah.es/en/metab/enzimas/inicio.htm) fitting kinetic models to experimental data, and Sigma Plot 12.0. ICx values were calculated using the Excel add-in ED50V10 and STATISTICA 12 PL software package.

## 4. Conclusions

*P. moorei* KB4 exhibits metabolic activity and the ability to grow in a wide range of non-optimal conditions and in the presence of xenobiotics. It is capable of adaptation to changing environmental conditions. These features make it very valuable for potential application in wastewater treatment plants. The toxicity analysis showed a protective effect of the carrier at low paracetamol concentrations, and a pronounced phenomenon of hormesis was observed in the immobilised systems. The kinetic analysis of the paracetamol degradation process shows a decrease in the degradation activity of the immobilised strain KB4. Cells free without glucose and immobilised with glucose degraded the drug with a significant delay. Although the results show that the strain KB4 is a weak biofilm producer, the obtained biofilm is sufficiently stable on the medium, which allows the use of the preparation obtained in this way for up to 10 degradation cycles of 20 mg/L paracetamol.

It may be concluded that the capacity of the formation of a biofilm with high metabolic activity by the strain KB4, the short immobilisation time in non-demanding conditions, its paracetamol degradation and its improved tolerance of immobilised cells make this strain suitable for more complicated biodegradation goals.

## Figures and Tables

**Table 1:** Parameters V_max_ (mg/L*h), K_S_ (mg/L) and h of the Hill equation, representing the dependence of the paracetamol degradation rate on its initial concentration for specific degradation settings.

Reaction Setting	*V*_max_ (mg/L*h)	K_S_ (mg/L)	h
Free cells without co-substrate	21.08 ± 3.69	93.81 ± 22.61	1.61 ± 0.22
Free cells with 1 g/L glucose	30.70 ± 6.80	289.59 ± 88.37	1
Immobilised cells without co-substrate	15.15 ± 2.45	145.39 ± 37.10	1
Immobilised cells with 1 g/L glucose	6.86 ± 0.43	37.84 ± 3.38	1.95 ± 0.27

**Table 2:** Toxicity of paracetamol to planktonic and immobilised *P. moorei* KB4 cells determined by the ATP method.

Time	2 h		4 h		24 h	
	Immobilised Cells	Free Cells	Immobilised Cells	Free Cells	Immobilised Cells	Free Cells
IC_05_	15.246	3.599	13.002	6.000	9.565	5.462
IC_50_	15.719	8.598	14.557	8.928	11.660	6.316
IC_95_	16.208	25.033	16.139	19.426	19.179	9.250

**Table 3:** Toxicity of paracetamol to planktonic and immobilised *P. moorei* KB4 cells determined by the colony-forming units (CFU) method.

Time	24 h		24 h	
	Immobilised Cells	Free Cells	Immobilised Cells	Free Cells
Carbon Source	Paracetamol		Paracetamol with Glucose 1 g/L	
IC_05_	5.314	2.426	0.861	0.636
IC_50_	8.989	5.32	2.859	1.178
IC_95_	21.711	15.362	5.765	2.323

## Data Availability

The data presented in this study are available in this manuscript.

## Sample Availability

Strain KB4 is available from the authors.
